# Mannan Oligosaccharide Enhanced the Growth Rate, Digestive Enzyme Activity, Carcass Composition, and Blood Chemistry of Thinlip Grey Mullet (*Liza ramada*)

**DOI:** 10.3390/ani11123559

**Published:** 2021-12-14

**Authors:** Fawzy I. Magouz, Mohamed I. Bassuini, Malik M. Khalafalla, Ramy Abbas, Hani Sewilam, Salama Mostafa Aboelenin, Mohamed Mohamed Soliman, Asem A. Amer, Ali A. Soliman, Hien Van Doan, Mahmoud A. O. Dawood

**Affiliations:** 1 Animal Production Department, Faculty of Agriculture, Kafrelsheikh University, Kafr El-Sheikh 33516, Egypt; fawzymagouz25@gmail.com (F.I.M.); mohamedkamel201210@gmail.com (M.I.B.); ramyagri84@gmail.com (R.A.); Mahmoud.dawood@agr.kfs.edu.eg (M.A.O.D.); 2 Department of Aquaculture, Faculty of Aquatic and Fisheries Sciences, Kafrelsheikh University, Kafr El-Sheikh 33516, Egypt; malikkhalafalla@yahoo.com; 3 The Center for Applied Research on the Environment and Sustainability, The American University in Cairo, Cairo 11835, Egypt; sewilam@aucegypt.edu; 4 Department of Engineering Hydrology, RWTH Aachen University, 52062 Aachen, Germany; 5 Biology Department, Turabah University College, Taif University, Taif 21944, Saudi Arabia; s.aboelenin@tu.edu.sa; 6 Clinical Laboratory Sciences Department, Turabah University College, Taif University, Taif 21944, Saudi Arabia; mmsoliman@tu.edu.sa; 7 Central Laboratory for Aquaculture Research, Abbassa, Sakha Aquaculture Research Unit, Kafr El-Sheikh 33516, Egypt; asemamer1982@gmail.com; 8 Fish Nutrition Laboratory, Aquaculture Division, National Institute of Oceanography and Fisheries, Alexandria 11865, Egypt; Doctor.ali.soliman@gmail.com; 9 Department of Animal and Aquatic Sciences, Faculty of Agriculture, Chiang Mai University, Chiang Mai 50200, Thailand; 10 Science and Technology Research Institute, Chiang Mai University, Chiang Mai 50200, Thailand

**Keywords:** mullet, feed utilization, antioxidant enzyme system, feed additives, proximate chemical composition

## Abstract

**Simple Summary:**

Sustainable aquaculture requires natural alternative substances with high potential in enhancing the performance and wellbeing of aquatic animals. In this regard, the present study tested the possibility of using mannan oligosaccharides (MOS) in the diets of grey mullet as functional additives. For 8 weeks, fish were fed with enriched diets containing 0, 0.5, 1, and 2% MOS. The results showed marked improvements in the growth performance, digestive enzyme activity, blood chemistry, and antioxidative capacity. In conclusion, dietary MOS at 0.5–1% is required to enhance the productivity of grey mullet.

**Abstract:**

Mannan oligosaccharide (MOS) is prebiotic with high functionality in aquaculture. The current study investigated the potential roles of MOS on the growth performance, digestive enzyme activity, carcass composition, and blood chemistry of Thinlip grey mullet (*Liza ramada*). Four tested diets with 34.49% crude protein and 6.29% of total lipids were prepared and fortified with 0, 0.5, 1, and 2% MOS. Fish of initial weight = 5.14 ± 0.11 g/fish were distributed in 12 hapas (0.5 × 0.5 × 1 m) at 15 fish per hapa (triplicates) and fed the test diets to the satiation level two times a day (08:00 and 15:00) for eight weeks. At the end of the trial, all fish were weighed individually for growth performance calculation. Blood was collected to check blood chemistry traits, and intestines were dissected for digestive enzyme analysis. Fish treated with MOS had marked enhancement in the final body weight, feed conversion ratio, protein gain, and protein retention regardless of inclusion dose (*p* < 0.05). The weight gain, specific growth rate, and protein efficiency ratio were meaningfully enhanced by including MOS at 0.5 and 1%, followed by fish fed with 2% MOS, while the lowest values were in the control group (*p* < 0.05). Insignificant influences of MOS were seen on the chemical composition of carcass components (moisture, crude protein, total lipids, and ash) (*p* > 0.05). Fish treated with MOS at 0.5 and 1% had marked enhancement in the amylase, lipase, and protease activities regardless of inclusion dose (*p* < 0.05). The blood total protein and albumin levels were meaningfully enhanced by including MOS at 0.5 and 1%, followed by fish fed with 2% MOS, while the lowest values were in the control group (*p* < 0.05). The blood globulin was significantly enhanced in fish fed 1% MOS than fish treated with 0, 0.5, and 2% of MOS (*p* < 0.05). The blood lysozyme activity was meaningfully enhanced by including MOS at 1%, followed by fish treated with 0.5 and 2%, while the lowest values were in the control group (*p* < 0.05). Phagocytic activity and phagocytic index were markedly improved in fish treated with 1 and 2% MOS, followed by those fed 0.5% compared with fish fed MOS-free diet (*p* < 0.05). Superoxide dismutase and glutathione peroxidase were markedly improved in fish treated with 1, and 2% MOS, followed by those fed 0.5% compared with fish fed MOS-free diet (*p* < 0.05). Dietary MOS (0.5, 1, and 2%) meaningfully enhanced catalase activity while decreased the malondialdehyde concentration (*p* < 0.05). In summary, dietary MOS is required at 0.5–1% for enhancing the growth rate, feed efficiency, digestive enzyme activity, blood chemistry, and antioxidative capacity of grey mullet.

## 1. Introduction

Aquaculture is a vital sector for sustaining food security for humanity [1]. The expansion of aquaculture activity is strongly correlated with consumer needs considering the available resources [2]. Grey mullets are a valuable tasty fish species in several countries, including Egypt, to increase fish diversity for consumers [3]. Mullets can grow in a wide range of salinities in tropical and subtropical conditions as low trophic level fish (2.3; www.fishbase.org (accessed on 1 May 2021)) [4], making them a reasonable fish species for sustainable aquaculture activity [5]. Growing fish in such stressful conditions requires water, management, handling, and feed qualities [6]. Ensuring optimum rearing conditions is the main factor for maximizing the productivity of finfish species [7]. However, unstable environmental features may deteriorate feeding habits, health status, and tolerance to infectious diseases [8].

New farming strategies suggest functional additives as friendly alternatives for chemotherapies to enhance the production and welfare of finfish species [9]. Several substances are used in aquaculture and validated as effective growth promotors, immunostimulants, and antioxidative agents [10,11]. Probiotics, prebiotics, medicinal herbs, and immunostimulants are commonly included in aquafeed to enhance aquatic animals’ performances and well-being [12,13]. Prebiotics are indigestible substances produced via the fermentation of yeast cell walls and functional carbohydrates [14,15]. Mannan oligosaccharides (MOS) are active prebiotic additives known for their functionality as enhancers for intestinal digestion and immunity with an apparent antibacterial capacity [16,17]. MOS is yeast-derived glucomannoprotein complexes showing several biological effects such as leucocyte activation and upregulation of proinflammatory cytokines [18,19]. In this context, Ringø, et al. [20] reported that mannose-containing molecules provoke intracellular signaling associated with proinflammatory cytokines production that may enhance aquatic animals’ immunity and well-being. Dietary MOS was investigated in several fish species and resulted in enhanced growth performance [21], digestion capacity [22], intestinal health [23,24], immune response [25,26], antioxidative status [27], and resistance against farming stressors [28,29]. The efficiency of MOS depends mainly on the fish species, fish size, duration of feeding, doses of inclusion, and culture conditions [30]. Thus, additives inclusion based on the species-specific manner is necessary to approve MOS for finfish species.

Thinlip grey mullet (*Liza ramada*) is a commercially farmed fish with a high market value in the Egyptian market [31]. This would make grey mullet incredibly sustainable, even better than common carp (*Cyprinus carpio*), and similar to Nile tilapia (*Oreochromis niltoticus*) due to lower protein requirements and lower need for animal-based feed ingredients [4]. Thus, more efforts are required to enhance productivity considering the beneficial role of MOS. Although dietary MOS is effectively applied in several finfish species, no studies investigated the functionality of MOS in grey mullet. In this sense, the current study was planned to test the potential effects of MOS on the growth rate, feed efficiency, blood immunity of grey mullet.

## 2. Materials and Methods

### 2.1. Trial Conditions

Experimental diets were prepared by mixing the dry ingredients (fish and soybean meals, wheat bran, yellow corn, rice bran, and mineral and vitamin mixture) (Table 1). The dry powder of ingredients was divided into four portions then mixed with mannan oligosaccharides (Bio-Mos^®^, Alltech, Inc., Lexington, KY, USA) at 0, 0.5, 1, and 2%. Fish oil was added and well mixed with the prepared diets. Afterward, water was included and mixed with the diets before pelleting with the laboratory pelleting machine. A dough of 1–2 mm pellets prepared and dried in a lab oven at 50 °C. The prepared diets were then stocked in plastic bags and put in the refrigerator till used in the trial. The chemical composition of the basal diet was confirmed by following the standard method [32].

Three hundred juveniles of Thinlip grey mullet (*Liza ramada*) were obtained from Bughaz El-Burullus (Lake Burullus), located on the coast of the Mediterranean Sea (Baltim city, Kafr El-sheikh governorate, Egypt). Fish were gently moved to the Fish Nutrition Laboratory, Baltim Unit, National Institute of Oceanography and Fisheries and stocked in outdoor concrete tanks (3 × 2 × 1.7 m) under ambient light conditions. In the concrete tanks, water was running in a flow-through system with average values of 27.21 ± 0.23 °C, 7.2 ± 0.4, 6.3 ± 0.33 mg/L, and 0.21 ± 0.02 mg/L for temperature, pH, dissolved oxygen, and total ammonia, respectively. Fish fed the basal diet for 14 days before distributing among the experimental units. Then, fish of similar initial weight 5.14 ± 0.11 g were individually weighed and distributed in 12 hapas (0.5 × 0.5 × 1 m) at 15 fish per hapa. Twenty fish from the stock were collected, stunned, washed with fresh water, and kept at −20 °C for the initial body chemical composition. All hapas were fixed in one concrete tank with a set of water inlets and outlets. Fish were fed the diets up to apparent satiation level two times a day (08:00 and 15:00). The amount of consumed feed was recorded regularly during the trial to calculate the total feed intake. We have weighed the fish every 2 weeks to follow up on the growth performance and health status during the study. Once marked differences between fish fed MOS and the control were detected in terms of the final body weight, the trial was terminated. The water quality indices were checked throughout the trial and recorded. The water temperature, pH, dissolved oxygen, and total ammonia were 27.32 ± 0.41 °C, 7.11 ± 0.3, 5.83 ± 0.41 mg/L, and 0.21 ± 0.01 mg/L, respectively.

### 2.2. Final Sampling

After eight weeks, all fish fasted for 24 h before the final sampling. Then all fish were anesthetized with tricaine methanesulphonate (MS-222; 25 mg/L), weighed, and counted to calculate the growth-related indices using the following Equations:

WG (%) = 100 × ((FBW − IBW)/IBW)

SGR (%/day) = 100 × (ln FBW (g) − ln IBW (g))/number of days

FCR = FI/(FBW − IBW)

PER = (FBW − IBW)/dry protein intake (g)

PG (g/kg weight gain) = {(FBW × final whole body protein content (%)/100) − (IBW × initial whole body protein content (%)/100)}/(WG) × 1000

PR (% of intake) = (protein gain (g/kg weight gain) × 100)/protein intake (g/kg weight gain)

Survival (%) = 100 × FN/IN

IBW and FBW were the initial and final body weight (g) of fish, respectively; IN and FN were the initial and final fish. The weight gain (WG), specific growth rate (SGR), feed conversion ratio (FCR), total dry feed intake (FI), the protein efficiency ratio (PER), protein gain (PG), and protein retention (PR).

Then, after being anesthetized with MS-222, five fish were randomly selected from each hapa followed by chilling in ice/water slurry until fish die, weighed, and immediately kept at −20 °C for the whole-body chemical analysis [34]. Fish were then dried and crushed into powder form. The diets and fish whole body were analyzed for moisture, crude protein, total lipid, and ash in triplicate, using standard methods [32]. The moisture content was evaluated following oven drying (Memmert UN110, Buchenbach, Germany) at 105 °C until a constant dry weight was reached. The ash content was determined using a muffle furnace (Heraeus Instruments K1252, Hanau, Germany) at 550 °C for 6 h. Crude protein was analyzed using the Micro-Kjeldahl apparatus (Foss Kjeltec 2200, Hillerqd, Denmark). Total lipid content was determined by petroleum ether extraction in the Soxhlet apparatus for 6 h.

Another three fish per hapa (nine fish per treatment) were gently bled from the caudal vein using 2.5 mL heparinized syringes to collect blood for phagocytosis analysis. Besides using non-heparinized syringes, blood was collected for serum separation. Samples were left for 4 h at 4 °C, then centrifuged at 3000× *g* for 15 min under 4 °C for serum collection. The collected blood or serum samples from each hapa (three fish) were divided in two Eppendorf (1.5 mL) (whole blood and serum) and kept for further analysis. Serum samples were kept at −80 °C for further biochemical analysis. Besides, three fish per hapa (9 fish per treatment) were killed and dissected, and their intestines were extracted to detect the activity of digestive enzymes.

### 2.3. Digestive Enzyme Activity 

The homogenate was prepared by rinsing the intestines in ice-cold phosphate-buffered saline (PBS) (pH 7.5; 1 g per 10 mL). It was then homogenized and centrifuged at 7168× *g* for 5 min, and the supernatant was collected from three fish per hapa and stored at 4 °C for further analysis. The total protein content was measured using diluted homogenates following Lowry, et al. [35] using bovine serum albumin as a standard. Protease activity was evaluated by following Anson [36] using Folin phenol reagent, and amylase activity was measured according to the methods of Jiang [37] and Worthington [38] using iodine solution to reveal non-hydrolyzed starch. Protease and amylase activity were expressed as specific activity (units per mg of protein). The specific activity of lipase was assessed based on previously described protocols by Borlongan [39] and Jin [40] with olive oil as a substrate. Fatty acids derived from enzymatic hydrolysis of triglyceride in a stable emulsion of olive oil were titrated with NaOH. One unit of specific activity of lipase was determined as the volume of 0.05 N NaOH needed to neutralize fatty acid release after 6 h of incubation with the substrate. Lipase activity was expressed as units per gram of intestinal content.

### 2.4. Blood Analysis

Serum total proteins and albumins were determined, according to Doumas, et al. [41] and Dumas and Biggs [42]. Serum aspartate aminotransferase (AST), alanine aminotransferase (ALT), creatinine, urea, triglycerides, and total cholesterol were detected by RA-50 chemistry analyzer (Diagnostics Manufacturing Limited, Bayer, Dublin, Ireland) using readymade chemicals (kits) supplied by Pasteur labs, France, following the manufacturer’s instructions. 

Leukocyte phagocytic function followed the method of Cai, et al. [43]. The number of leukocytes that engulfed bacteria was counted as percentages in relation to the total leukocyte number in the smear from the phagocytosis assay. By following Kawahara, et al. [44], the phagocytic activity and phagocytic index were determined. Analysis of serum lysozyme activity was performed using a turbidimetric assay, according to Ellis, et al. [45].

Superoxide dismutase (SOD), catalase (CAT), and glutathione peroxidase (GPx) in serum were measured using diagnostic reagent kits following the manufacturer’s (Biodiagnostic, Dokki, Giza, Egypt) instructions. The concentration of malondialdehyde (MDA) was detected by following Uchiyama and Mihara [46] and expressed as nmol MDA/g.

### 2.5. Statistical Analysis

Shapiro–Wilk and Levene tests confirmed normal distribution and homogeneity of variance. The mean values of blood analysis and digestive enzyme activity of three fish per hapa were calculated then the statistical analysis was made using the hapa as a statistical unit. The obtained data were subjected to one-way ANOVA. Differences between means were tested at *p* < 0.05 level using the Duncan test as a post-doc test. All the statistical analyses were done via SPSS version 22 (SPSS Inc., Chicago IL, USA).

## 3. Results

### 3.1. Growth Performance and Carcass Composition

The growth-related indices of grey mullet treated with MOS are presented in Table 2. Fish treated with MOS had marked enhancement in the final body weight, feed conversion ratio, protein gain, and protein retention regardless of inclusion dose (*p* < 0.05). The weight gain, specific growth rate, and protein efficiency ratio were meaningfully enhanced by including MOS at 0.5 and 1%, followed by fish treated with 2%, while the lowest values were in the control group (*p* < 0.05). Insignificant influences of MOS were seen on the survival rate of grey mullet treated with MOS (*p* > 0.05). Similarly, the chemical characteristics of carcass components were not meaningfully impacted by dietary MOS (*p* > 0.05) (Table 3).

### 3.2. Digestive Enzyme Activity

**Figure 1:**
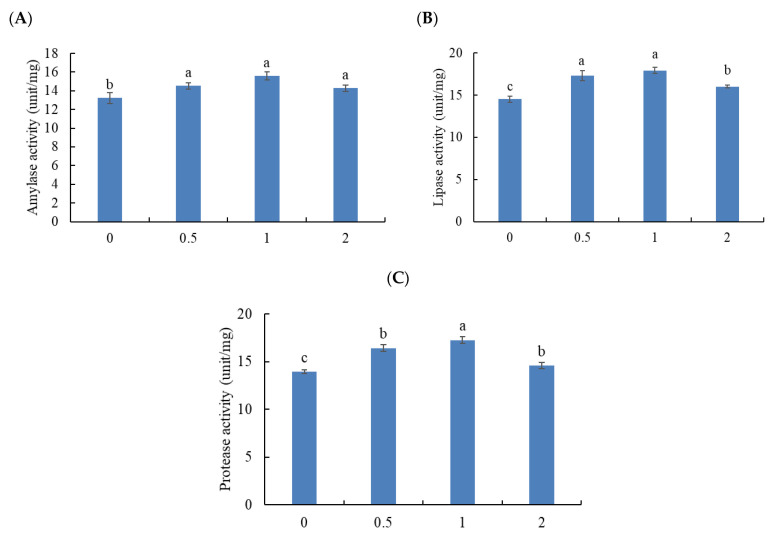
Digestive enzyme activity: (**A**) amylase, (**B**) lipase, and (**C**) protease activities of Thinlip grey mullet fed dietary mannanoligosaccharide for 8 weeks. Bars present means ± S.E. and different letters are significantly different (*p* < 0.05) (*n* = 3).

Fish treated with MOS had marked enhancement in the amylase activity regardless of inclusion dose (*p* < 0.05) (Figure 1A). The lipase activity was meaningfully enhanced by including MOS at 0.5 and 1%, followed by fish treated with 2%, while the lowest values were in the control group (*p* < 0.05) (Figure 1B). The highest protease activity was seen in fish fed 1% MOS followed by those treated with 0.5 and 2%, while the lowest protease activity was seen in fish fed the basal diet (*p* < 0.05) (Figure 1C).

### 3.3. Biochemical Blood Indices

No marked effect of dietary MOS was seen on the blood bio-indices except for the blood metabolites (*p* > 0.05) (Table 4). The blood total protein and albumin levels were meaningfully enhanced by including MOS at 0.5 and 1%, followed by fish treated with 2%, while the lowest values were in the control group (*p* < 0.05) (Table 4). The blood globulin was significantly enhanced in fish fed 1% MOS when compared to fish treated with 0, 0.5, and 2% of MOS (*p* < 0.05) (Table 4).

### 3.4. Immune Blood Parameters

**Figure 2:**
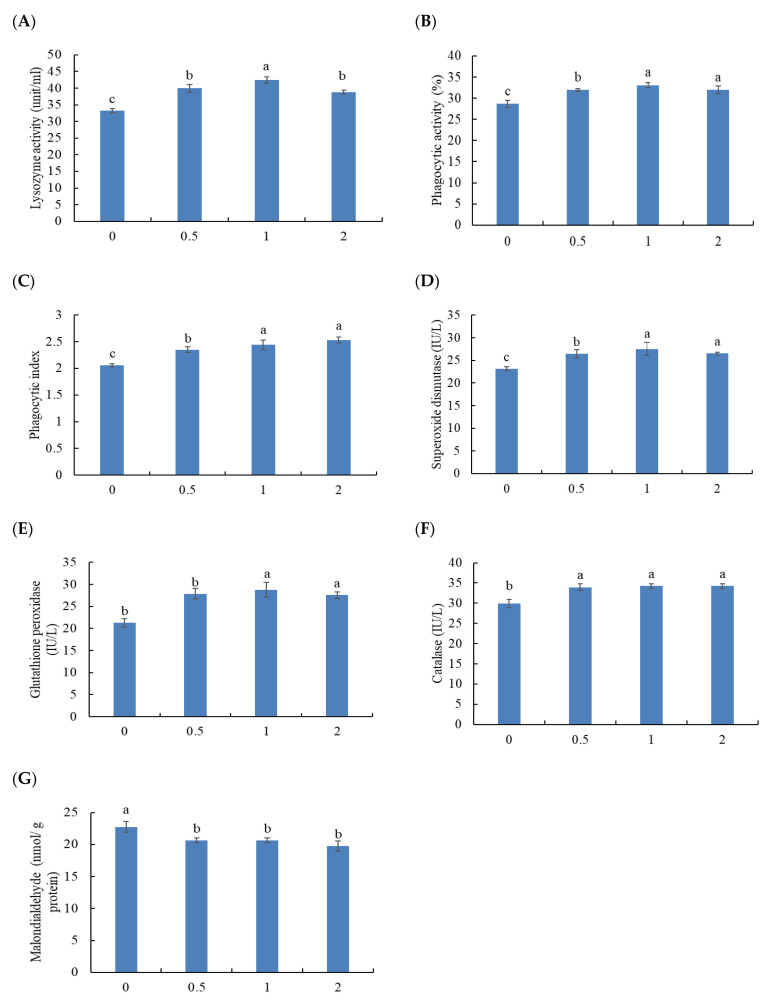
Blood immune parameters: (**A**) lysozyme activity, (**B**) phagocytic activity, (**C**) phagocytic index, (**D**) superoxide dismutase, (**E**) glutathione peroxidase, (**F**) catalase, and (**G**) malondialdehyde level of Thinlip grey mullet fed dietary mannanoligosaccharide for 8 weeks. Bars present means ± S.E. and different letters are significantly different (*p* < 0.05) (*n* = 3).

The blood lysozyme activity was meaningfully enhanced by including MOS at 1%, followed by fish treated with 0.5 and 2%, while the lowest values were in the control group (*p* < 0.05) (Figure 2A). Phagocytic activity and phagocytic index were markedly improved in fish treated with 1 and 2% MOS, followed by those fed 0.5% compared with fish fed MOS-free diet (*p* < 0.05) (Figure 2B,C).

Superoxide dismutase and glutathione peroxidase were markedly improved in fish treated with 1 and 2% MOS, followed by those fed 0.5% compared with fish fed MOS-free diet (*p* < 0.05) (Figure 2D,E). Dietary MOS meaningfully enhanced catalase activity while decreased the malondialdehyde concentration compared with fish fed the basal diet (*p* < 0.05) (Figure 2F,G).

## 4. Discussion

The inclusion of active substances in aquafeed is effectively applied in aquaculture to enhance aquatic animals’ performances and well-being [8,47]. Prebiotic-related additives resulted in positive effects on the productivity of finfish species, making them friendly alternatives for chemical drugs [48]. Many studies showed the direct effect of MOS on enhancing the growth behavior and health status, but others indicated no marked influences of using MOS on finfish species [23,24,49]. For this reason, the inclusion of MOS in aquafeed has to be evaluated based on the species-specific manner. 

The results showed that grey mullet fed dietary MOS had enhanced growth performance which agrees with most of the studies investigating the effect of MOS on the growth performance of finfish species [21,49]. In this context, Piccolo, et al. [50] and Dimitroglou, et al. [51], who indicated that MOS did not influence the growth performance of sharpsnout sea bream (*Diplodus puntazzo*) and gilthead sea bream (*Sparus aurata*). The obtained results show that the effects are diverse and can be species-specific effects. The differences in growth performance are related to the feeding habits, life stage, feeding duration, and fish species [52]. The role of MOS in improving the growth performance of fish is associated with the MOS capacity of enhancing feed utilization and digestion of nutrients [30]. Markedly the results showed enhanced feed efficiency in the intestines of grey mullets illustrating that the high feed intake is the main reason for high growth performance. MOS is one of the yeast cell wall derivatives with high polypeptides, vitamins, and active proteins [53]. The modern concept of prebiotics implies the use of selective compounds to favor the growth of the protective indigenous gut microbiota [9,54]. Beneficial microorganisms are responsible for facilitating the digestion and absorption of nutrients in fish intestines by producing digestive enzymes [20]. Concurrently, the activation of the digestive enzymes by dietary MOS led to high feed utilization and growth performance [55].

The enhancement of the growth performance of Thinlip grey mullet fed dietary MOS is most likely also related to the improved digestive enzyme activity. The detection of digestive enzyme activities is essential for feeding with specific diet formulation on feed utilization [56,57]. High digestion and absorption capacity in fish intestines could be related to the role of intestinal microbiota to release nutrients into the bloodstream by crossing the intestinal barrier [9]. Our results showed enhanced amylase, lipase, and protease activities in grey mullet, which agreed with Akter, et al. [58] and Wu, et al. [59], who stated that striped catfish (*Pangasianodon hypophthalmus*) and yellow catfish (*Pelteobagrus fulvidraco*) fed dietary MOS had enhanced digestive enzyme activities. The improvements in the FCR, protein efficiency ratio, protein gain, and protein retention are strongly related to the effect of MOS on improving the feed efficiency of grey mullet. Further, enhanced feed utilization could explain the increased growth performance of grey mullet-fed dietary MOS.

Measuring biochemical blood indices helps diagnose the feed utilization quality, metabolic function, immune response, and stress resistance of aquatic organisms [60]. The protein metabolites (total protein, albumin, and globulin), lipid metabolites (total cholesterol and triglycerides), liver condition (ALT and AST), and kidney condition (creatinine and urea) related indices [61] are the main factors detected in the present study in response with MOS feeding in grey mullet. The results showed no marked effects on the measured indices except for blood protein metabolites which increased meaningfully in grey mullet treated with dietary MOS. The results indicate that dietary MOS had beneficial side effects on the blood proteins without impacting liver and kidney functions [62]. Improved blood proteins are consistently correlated with high digested nutrients, hormones, enzymes, and immune metabolites in fish’s blood [63]. In a similar sense, Dawood, et al. [28] and Yuji-Sado, et al. [64] fed dietary MOS showed improved blood proteins without negative impacts on the hepato-renal function of red sea bream (*Pagrus major*) and Nile tilapia (*Oreochromis niloticus*), respectively.

Lysozyme and phagocytosis activities are non-specific immune responses involved in the protection against pathogenic invaders in the fish body [65]. Lysozyme can deactivate the peptidoglycan layer in the pathogenic bacterial cell walls leading to high resistance against infection [66]. Besides, the phagocytosis function is the tool of combating microbial infection via phagocytic activity [67]. The results showed activated lysozyme and phagocytic activities in grey mullet fed dietary MOS referring to high immunity. The results are in line with Dawood, et al. [28] and Ren, et al. [23], who reported increased lysozyme and phagocytic activities in red sea bream and hybrid grouper (*Epinephelus lanceolatus* ♂ × *E. fuscoguttatus* ♀) fed dietary MOS. The potential role of MOS on activating fish immunity is probably related to its effect on activating the immune cells [16]. The effect of MOS begins with activating the local intestinal immunity, which is correlated with the whole fish body immunity [68].

The antioxidant capacity of fish is another vital tool to defend the fish body from reactive oxygen metabolites (ROS) responsible for lipid peroxidation during infection and stress [69]. High ROS levels induce oxidative stress and impairment of cell function and can be indicated by detecting malondialdehyde (MDA) concentration [68]. Thus, in this study, high activities of related antioxidant enzymes (SOD, CAT, and GPx) with low levels of MDA indicate the positive role of MOS in maintaining the high wellbeing of grey mullet. The results are similar with Dawood, et al. [28] and Ren, et al. [23], who indicated that red sea bream and hybrid grouper-fed dietary MOS showed activated antioxidant capacity. The activation of antioxidative capacity resulting from MOS feeding correlates with MOS role in degenerating excessive ROS in the entire body [27].

The study was performed for only 60 days, while the effect of MOS should be investigated through the entire farming life of the fish. It has been reported that MOS effects on fish performance are vary depending on the duration of feeding. Most importantly, Terova, et al. [70] reported that the innate immune response of European sea bass (*Dicentrarchus labrax*) was activated by dietary MOS for 60 days. The authors claimed that MOS influence on the immune system depends on the feeding duration and dose of supplementation. They also concluded that a long feeding period (60 days) did not result in higher dicentracin transcript levels than after 30 days of feeding regardless of the dose supplementation. Therefore, further future studies are recommended to evaluate the effect of dietary MOS through the entire farming life of the fish.

## 5. Conclusions

The obtained results indicated that dietary MOS is required at 0.5–1% for enhancing the growth rate, feed efficiency, blood immunity, and antioxidative capacity of Thinlip grey mullet. Further studies are needed to understand the role of MOS on Thinlip grey mullet performances using molecular tools.

## Figures and Tables

**Table 1:** Basal diet and proximate chemical composition (%, on dry matter basis).

Ingredients	%	Chemical Composition	%
Fish meal	15	Crude protein	34.49
Soybean meal	40	Total lipids	6.29
Yellow corn	15	Ash	7.55
Gluten	7	Crude fibers	5.12
Wheat bran	12	Nitrogen free extract	46.55
Wheat flour	4.92	Gross energy (KJ/g) ^2^	18.63
Fish oil	3		
Vitamin and mineral mix ^1^	2		
Dicalcium phosphate	1		
Vitamin C	0.08		

^1^ Vitamin and mineral mixture detailed by Dawood, et al. [31]. The nitrogen-free extract was calculated by difference 100%—(total lipids + crude protein + ash + crude fibers). ^2^ Gross energy was calculated based on crude protein, total lipids, and nitrogen-free extract values as 23.6, 39.5, and 17.2 KJ/g, respectively [33].

**Table 2:** Growth performance of Thinlip grey mullet fed dietary mannanoligosaccharide.

Item	0.0%	0.5%	1.0%	2.0%
IBW (g)	5.13 ± 0.10	5.11 ± 0.02	5.13 ± 0.04	5.16 ± 0.02
FBW (g)	20.91 ± 0.31 ^b^	23.93 ± 0.37 ^a^	23.62 ± 0.53 ^a^	22.64 ± 0.12 ^a^
WG (%)	307.68 ± 10.03 ^c^	368.26 ± 6.77 ^a^	360.10 ± 7.67 ^a^	339.26 ± 4.26 ^b^
SGR (%/day)	2.34 ± 0.04 ^c^	2.57 ± 0.02 ^a^	2.54 ± 0.03 ^a^	2.47 ± 0.02 ^b^
FI (g/fish)	22.67 ± 1.84	22.89 ± 0.02	22.42 ± 0.44	21.64 ± 0.02
FCR	1.43 ± 0.10 ^a^	1.22 ± 0.02 ^b^	1.21 ± 0.03 ^b^	1.24 ± 0.01 ^b^
PER	2.32 ± 0.15 ^c^	2.71 ± 0.05 ^a^	2.75 ± 0.09 ^a^	2.66 ± 0.03 ^b^
PG	131.60 ± 5.00 ^b^	143.73 ± 1.15 ^a^	143.39 ± 2.51 ^a^	143.92 ± 1.53 ^a^
PR	19.49 ± 2.17 ^b^	20.71 ± 0.18 ^a^	21.36 ± 0.61 ^a^	21.87 ± 0.24 ^a^
Survival (%)	97.78 ± 2.22	100.00 ± 0.00	100.00 ± 0.00	97.78 ± 2.22

Means ± S.E. in the same row with different letters differ significantly (*p* < 0.05). Where IBW and FBW were initial and final body weight (g) of fish, respectively. Weight gain (WG), specific growth rate (SGR), feed conversion ratio (FCR), total dry feed intake (FI), the protein efficiency ratio (PER), protein gain (PG), and protein retention (PR).

**Table 3:** Carcass composition (% of fresh matter) of Thinlip grey mullet fed dietary mannanoligosaccharide.

Item	Initial Body Composition	0.0%	0.5%	1.0%	2.0%
Moisture	80.21 ± 0.55	78.18 ± 0.55	76.97 ± 0.41	77.08 ± 0.13	76.75 ± 0.12
Crude protein	12.12 ± 0.38	13.42 ± 0.38	14.34 ± 0.09	14.31 ± 0.04	14.35 ± 0.12
Total lipid	3.83 ± 0.05	4.13 ± 0.05	4.61 ± 0.03	4.84 ± 0.14	4.88 ± 0.13
Ash	3.35 ± 0.09	3.69 ± 0.09	3.56 ± 0.06	3.60 ± 0.14	3.94 ± 0.09

Means ± S.E. in the same row without different letters, non-significantly differ (*p* > 0.05).

**Table 4:** Blood biochemical indices of Thinlip grey mullet fed dietary mannanoligosaccharide.

Item	0.0%	0.5%	1.0%	2.0%
ALT (U/I)	3.35 ± 0.12	3.25 ± 0.13	3.25 ± 0.08	3.27 ± 0.24
AST (U/I)	74.81 ± 1.72	74.07 ± 1.15	73.82 ± 1.32	74.92 ± 1.46
Total protein (g/dl)	4.13 ± 0.12 ^c^	4.46 ± 0.21 ^a^	4.55 ± 0.18 ^a^	4.30 ± 0.11 ^b^
Albumin (g/dl)	2.17 ± 0.08 ^c^	2.53 ± 0.11 ^a^	2.43 ± 0.13 ^a^	2.37 ± 0.14 ^b^
Globulin (g/dl)	1.96 ± 0.09 ^b^	1.93 ± 0.05 ^b^	2.12 ± 0.08 ^a^	1.93 ± 0.04 ^b^
Creatinine (mg/dl)	0.27 ± 0.02	0.25 ± 0.01	0.24 ± 0.02	0.23 ± 0.01
Urea (mg/dl)	4.87 ± 0.21	4.71 ± 0.11	4.61 ± 0.12	4.52 ± 0.21
Total cholesterol (mg/dl)	87.33 ± 2.72	92.00 ± 2.15	97.00 ± 2.69	90.18 ± 2.66
Triglycerides (mg/dl)	133.83 ± 3.24	145.33 ± 4.88	142.00 ± 4.23	136.67 ± 3.91

Means ± S.E. in the same row with different letters differ significantly (*p* < 0.05) (*n* = 3). Serum aspartate aminotransferase (AST) and alanine aminotransferase (ALT).

## Data Availability

Data available from the corresponding author at convenient request.
